# Prevalence and Associations of Steep Cornea/Keratoconus in Greater Beijing. The Beijing Eye Study

**DOI:** 10.1371/journal.pone.0039313

**Published:** 2012-07-06

**Authors:** Liang Xu, Ya Xing Wang, Yin Guo, Qi Sheng You, Jost B. Jonas, the Beijing Eye Study Group

**Affiliations:** 1 Beijing Institute of Ophthalmology, Beijing Tongren Hospital, Capital Medical University, Beijing, China; 2 Department of Ophthalmology, Medical Faculty Mannheim of the Ruprecht-Karls-University, Heidelberg, Germany; Wayne State University, United States of America

## Abstract

**Purpose:**

To evaluate the prevalence and associated factors of steep cornea/keratoconus in the adult Chinese population.

**Methods:**

The population-based Beijing Eye Study 2011 included 3468 individuals with a mean age of 64.6±9.8 years (range: 50–93 years). A detailed ophthalmic examination was performed including optical low-coherence reflectometry. Steep cornea/keratoconus were defined as an anterior corneal refractive power exceeding 48 diopters.

**Results:**

Mean refractive power of the cornea was 43.16±1.45 diopters (range: 36.51 to 48.46 diopters; flattest meridian) and 43.98±1.52 diopters (range: 37.00 to 52.88 diopters; steepest meridian). A steep cornea/keratoconus defined as corneal refractive power of ≥48 diopters and ≥49 diopters was detected in 27 subjects (prevalence rate: 0.9±0.2%) and 6 (0.2± 0.1%) subjects, respectively. Presence of steep cornea/keratoconus was associated with shorter axial length (*P*<0.001), smaller interpupillary distance (*P* = 0.038), lower best corrected visual acuity (*P* = 0.021), higher cylindrical refractive error (*P*<0.001) and more myopic refractive error (*P*<0.001). It was not significantly associated with gender, body height, psychic depression, cognitive function, blood concentrations of glucose, lipids, creatinine and C-reactive protein, blood pressure and quality of life score, nor with intraocular pressure, dry eye feeling, and lens thickness.

**Conclusions:**

A steep cornea/keratoconus defined as corneal refractive power of 48+ diopters has a prevalence of 0.9±0.2% among Chinese aged 50 years and above. Its prevalence was significantly associated with the ocular parameters of shorter axial length, smaller interpupillary distance, higher cylindrical and myopic refractive error and lower best corrected visual acuity, however, with none of the systemic parameters tested.

## Introduction

Keratoconus is a conical deformation of the anterior corneal surface leading to refractive myopia, irregular astigmatism and eventually to a marked reduction in visual acuity [1], [2]. Particularly in Western countries, it has been one of the most common reasons for keratoplasties [3]–[10]. Since there has so far been only one population-based study on the prevalence of keratoconus (or steep cornea) and since that study was performed in rural Central India on a semi-tribal population which may not be representative for other ethnic groups [11], we conducted the present study to examine the prevalence of keratoconus and steep cornea and its associations with other ocular or general parameters in a Chinese population. We defined a keratoconus/steep cornea as a corneal refractive power exceeding 48 diopters as measured by optical low-coherence reflectometry.

## Methods

### Ethics Statement

The Medical Ethics Committee of the Beijing Tongren Hospital approved the study protocol and all participants gave informed written consent, according to the Declaration of Helsinki.

The Beijing Eye Study 2011 is a population-based cross-sectional study in Northern China [12], [13]. It was carried out in 5 communities in the urban district of Haidian in the North of Central Beijing and in 3 communities in the village area of Yufa of the Daxing District south of Beijing. The only eligibility criterion for inclusion into the study was an age of 50+ years. In 2011, the 8 communities had a total population of 4403 individuals aged 50 years or older. In total, 3468 individuals (1963 (56.6%) women) participated in the eye examination, corresponding to an overall response rate of 78.8%. The study was divided into a rural part (1633 (47.1%) subjects; 943 (57.7%) women) and an urban part (1835 (52.9%) subjects; 1020 (55.6%) women). The mean age was 64.6±9.8 years (median, 64 years; range, 50–93 years).

All examinations were carried out in the communities, either in schoolhouses or in community houses. All study participants underwent an interview with standardized questions on their family status, level of education, income, quality of life, psychic depression, physical activity, known major systemic diseases such as arterial hypertension and diabetes mellitus, and quality of vision. Cognitive function was assessed using the MMSE (mini mental state examination) scale [14]. Fasting blood samples were taken for measurement of blood lipids, glucose and glycosylated hemoglobin HbA1c. Blood pressure was measured. Body height and weight and the circumference of the waist and hip were recorded. The ophthalmic examination included measurement of presenting visual acuity and uncorrected visual acuity. Best corrected visual acuity was assessed by automatic refractometry (Auto Refractometer AR-610, Nidek Co., Ltd, Tokyo, Japan). If uncorrected visual acuity was lower than 1.0, we additionally performed subjective refractometry. Intraocular pressure was measured by pneumotonometry by an experienced ophthalmologist. A slit lamp examination carried out by an ophthalmologist assessed lid abnormalities, Meibomian gland dysfunction, corneal disorders, and peripheral anterior chamber depth using van Herick’s method. The anterior segment was measured by slit-lamp adapted optical coherence tomography (OCT) (Heidelberg Engineering Co., Dossenheim, Germany). Using optical low-coherence reflectometry (Lensstar 900® Optical Biometer, Haag-Streit, 3098 Koeniz, Switzerland), biometry of the right eyes (or of the left eyes if measurements of the right eye were not possible) was performed for measurement of the anterior corneal curvature, central corneal thickness, anterior chamber depth, lens thickness and axial length. The pupil was dilated using tropicamide once or twice, until the pupil diameter was at least 6 mm. A second slit lamp assisted biomicroscopy searched for pseudoexfolaition syndrome. Digital photographs of the cornea and lens were taken using the slit lamp digital camera (Type BG-4, Topcon Medical Systems, Inc., Tokyo, Japan), and retro-illuminated photographs of the lens were obtained using the Neitz CT-R camera (Neitz Instruments Co., Tokyo, Japan). Monoscopic photographs of the macula and optic disc were taken using a fundus camera (Type CR6–45NM, Canon Inc. U.S.A.).

Using the biometric measurements of the anterior corneal curvature, the corneal refractive power was calculated assuming a refractive index of the cornea of 1.3315 as proposed by Olsen [15]. A steep cornea/keratoconus was defined as a corneal refractive power of equal to or higher than 48 diopters in the steepest corneal meridian. Selection criterion for the present study was the availability of corneal refractive power measurements.

Statistical analysis was performed using a commercially available statistical software package (SPSS for Windows, version 19.0, IBM-SPSS, Chicago, IL). In a first step, we examined the mean values (presented as mean±standard deviation) of the systemic and ocular parameters in the steep cornea/keratoconus group versus the non-keratoconus group. The Gaussian distribution of the parameters was tested using the Kolmogorov-Smirnov test. We calculated the significance of the differences between both groups using the student-t-test for non-paired samples for the parametric parameters, and the Mann-Whitney-U test for non-paired samples for the non-parametric parameters. In a second step, we performed a binary logistic regression analysis with the presence of steep cornea/keratoconus as dependent parameter and those parameters as independent parameters which were significantly associated with steep cornea/keratoconus in univariate analysis. 95% Confidence intervals (CI) and odds ratios (OR) were presented. All *P*-values were 2-sided and were considered statistically significant when the values were less than 0.05.

## Results

Out of the 3468 participants, keratometric measurements were available for 3166 (91.3%) subjects (1792 (56.6%) women). The mean age was 64.2±9.8 years (median: 63 years; range: 50 to 93 years), the mean refractive error (spherical equivalent) was −0.19±2.13 diopters (median: 0.25 diopters; range: −22.0 to +13.5 diopters). The group of subjects without keratometric measurements as compared with the group of subjects with keratometric measurements was significantly (*P*<0.001) older did not vary significantly in gender (*P* = 0.994).

Mean corneal curvature was in the flattest meridian 7.69±0.26 mm (median: 7.69 mm; range: 6.84 mm to 9.08 mm) and in the steepest meridian 7.55±0.26 mm (median: 7.54 mm; range: 6.39 mm to 8.96 mm). Correspondingly, the mean refractive power of the cornea in the flattest meridian was 43.16±1.45 diopters (median: 43.11 diopters; range: 36.51 to 48.46 diopters) and it was in the steepest meridian 43.98±1.52 diopters (median: 43.97 diopters; range: 37.00 to 51.88 diopters). A steep cornea/keratoconus (defined as corneal refractive power of ≥48 diopters) was detected in 27 subjects. The prevalence rate was 0.9±0.2% (95%CI: 0.6, 1.2). A steep cornea/keratoconus with a corneal refractive power of ≥49 diopters was detected in 6 subjects resulting in a prevalence rate of 0.2±0.1% (95% CI: 0.1, 0.4). Two subjects showed a corneal refractive power of ≥50 diopters (prevalence rate: 0.06±0.05% 95% CI: 0.02, 0.2).

In the whole study population, 2 eyes had undergone perforating keratoplasty for corneal ulceration and trauma. Corneal refractive power in these 2 eyes ranged between 43.9 and 44.5 diopters. Cataract surgery had been performed in 162 subjects of the study population. Their mean corneal refractive power was 43.04±1.52 diopters (median: 43.14 diopters; range: 39.05 to 46.95 diopters) in the flattest meridian and 44.20±1.69 diopters (median: 44.11 diopters; range: 39.37 to 48.18 diopters) in the flattest meridian. In two of the eyes after cataract surgery, the corneal refractive power exceeded 48 diopters. The eyes after cataract surgery and the phakic eyes did not differ significantly in corneal refractive power (*P* = 0.32 for flattest meridian; *P* = 0.09 for steepest meridian).

In univariate analysis, the prevalence of steep cornea/keratoconus was significantly associated with female gender (*P* = 0.006), shorter body height (*P*<0.001), lower level of education (*P*<0.001) and lower income (*P*<0.001), rural versus urban region of habitation (*P* = 0.008), lower cognitive score (*P* = 0.029), higher psychic depression score (*P* = 0.012), shorter axial length (*P*<0.001), smaller pupil diameter (*P* = 0.031), shorter interpupillary distance (*P*<0.001), smaller central corneal thickness (*P* = 0.033), larger cylindrical refractive error (*P* = 0.014), and lower best corrected visual acuity (*P* = 0.004) (Table 1, 2). The prevalence of steep cornea/keratoconus was not significantly associated with age (*P* = 0.99), body mass index (*P* = 0.33), systolic (*P* = 0.23) or diastolic (*P* = 0.46) blood pressure, quality of life index ( *P* = 0.33), blood concentrations of glucose (*P* = 0.87), cholesterol (*P* = 0.84), creatinine (*P* = 0.51) and C-reactive protein (*P* = 0.92), smoking (*P* = 0.11) or alcohol consumption (*P* = 0.39), dry eye feeling (*P* = 0.15), intraocular pressure readings (*P* = 0.37), lens thickness (*P* = 0.12) and optic disc size (*P* = 0.39) (Table 1,2).

**Table 1:** Demographic characteristics and ocular and systemic parameters of subjects with steep cornea/keratoconus (defined as a corneal refractive power of 48+ diopters) compared with the remaining population of the Beijing Eye Study 2011.

	Steep cornea/Keratoconus Group	Non-Steep Cornea/Keratoconus Group	*P*-Value
n	27	3139	
Gender (Males/Females)	4/23	1370/1769	0.006
Age (Years)	64.2±11.3	64.2±9.7	0.99
Body Height (cm)	155.0±6.2	161.9±8.1	<0.001
Body Weight (kg)	63.1±10.1	67.1±11.7	0.08
Body Mass Index (kg/m^2^)	26.3±4.0	25.6±3.8	0.33
Waist circumference(cm)	90.6±10.8	88.9±10.4	0.41
Hip circumference (cm)	100.3± 6.7	99.7±7.5	0.68
Syst. blood pressure (mmHg)	135.1± 23.4	130.2± 20.7	0.23
Diast. blood pressure (mmHg)	71.7±13.8	69.9±12.3	0.46
Heart pulse (beats/minute)	71.1±7.9	72.6±10.2	0.45
Level of Education (level)	3.2±1.2	3.9±1.1	<0.001
Income (level)	2.1±1.3	3.9±2.2	<0.001
Rural/urban habitation	20/7	1474/1665	0.008
Ever Smoking	18.5%	33.4%	0.11
Package Years	2.7±9.9	9.8±18.6	0.07
Alcohol Consumption	2.5±1.4	2.8±1.8	0.39
(Frequency Grade)			
Cognitive Score	24.9±3.6	26.4±3.6	0.03
Depression Score	25.2±7.6	22.5±5.2	0.01
Quality of Life Index	5.8±1.2	5.6±1,0	0.33

**Table 2:** Demographic characteristics and ocular and systemic parameters of subjects with steep cornea/keratoconus (defined as a corneal refractive power of 48+ diopters) compared with the remaining population of the Beijing Eye Study 2011.

	Steep cornea/Keratoconus Group	Non-Steep Cornea/Keratoconus Group	*P*-Value
Fasting blood examination			
High-density lipoproteins	1.52±0.52	1.48±0.44	0.90
(mmol/L)			
Low-density lipoproteins	3.44±0.86	3.37±0.92	0.74
(mmol/L)			
Triglycerides (mmol/L)	2.38±2.34	1.73±2.52	0.33
Glucose (mmol/L)	5.57±1.96	5.62±1.62	0.87
Cholesterol (mmol/L)	5.10±1.27	5.05±1.18	0.84
Creatinine (mmol/L) 28.24	64.11±12.40	67.92 ±	0.51
C-reactive protein (mg/L)	1.85±1.84	1.93±3.53	0.92
Axial Length (mm)	21.59±0.91	23.27±1.13	<0.001
Central Corn. Thickness (µm)	519±36	532±32	0.03
Corneal diameter (mm)	11.83±0.92	11.95±0.99	0.56
Anterior Chamber Depth (mm)	2.48±0.76	2.49±0.49	0.85
Lens Thickness (mm)	4.67±0.50	4.56±0.33	0.12
Pupil diameter (mm)	3.75±0.69	4.09±0.80	0.03
Pupil distance (mm)	57.46±7.36	61.85±4.45	<0.001
Refractive Error (Dpt)	-0.48± 1.89	-0.19±2.13	0.48
Spherical Refr. Error (Dpt)	-0.46± 1.59	-0.38± 2.03	0.84
Cylindrical Refr. Error (Dpt)	1.40±1.58	0.68±0.72	0.01
Best Corrected Visual Acuity	0.73±0.30	0.88±0.26	0.004
Intraocular pressure (mmHg)	15.0±3.7	14.5±2.7	0.37
Optic disc size (mm^2^)	2.71±0.56	2.59±0.51	0.39
Dry Eye Feeling (%)	57.7±9.9	43.6±0.9	0.15

The multivariate binary logistic regression analysis was carried out in several steps. A first step included the presence of steep cornea/keratoconus as dependent parameter and age and gender as independent parameters. It confirmed that the presence of steep cornea/keratoconus was significantly associated with female gender (*P* = 0.006), but not with age (*P* = 0.79). In a second step of the analysis with the additional parameters of body height and weight, only shorter body height (*P* = 0.001) was significantly associated with steep cornea/keratoconus, while age (*P* = 0.46), gender (*P* = 0.65) and body weight (*P* = 0.73) were not significantly associated. If body mass index was added, the result was similar. In a next step, the parameters of age, gender, body weight, or body mass index were dropped and the level of education was added. It revealed significant associations between the presence of steep cornea/keratoconus and lower body height (*P* = 0.001) and lower level of education (P = 0.04). If the region of habitation was added, prevalence of steep cornea/keratoconus was associated only with lower body height (*P*<0.001) and rural region (*P* = 0.04), while level of education was no longer significantly (*P* = 0.38) associated. In the next step of the statistical analysis, the parameter of package years of smoking was added. It showed that the presence of steep cornea/keratoconus was only associated with lower body height (*P* = 0.001) and rural region of habitation (*P* = 0.004), while package years of smoking (*P* = 0.152) was not significantly correlated with steep cornea/keratoconus. In a similar manner, the depression score (*P* = 0.10) and the cognitive score (*P* = 0.69) were not significantly associated with the presence of steep cornea/keratoconus. In a last step of the multivariate analysis, those ocular parameters were added which were significantly different between the steep cornea/keratoconus group and the non-keratoconus group in the univariate analysis. It revealed that after adjustment for body height and region of habitation, presence of steep cornea/keratoconus was significantly associated shorter axial length (*P*<0.001), smaller interpupillary distance (*P* = 0.038), lower best corrected visual acuity (*P* = 0.021), higher cylindrical refractive error (*P*<0.001) and more myopic refractive error (*P*<0.001) (Table 3). In that analysis, body height (*P* = 0.49) and area of habitation (*P* = 0.59) were no longer significantly associated with the prevalence of steep cornea/keratoconus.

**Table 3:** Associations between the presence of steep cornea/keratoconus (defined as a corneal refractive power of 48+ diopters) and systemic and ocular parameters in the Beijing Eye Study 2011.

Parameter	P-Value	Regression Coefficient	Odds Ratio	95% Confidence Interval
Axial length (mm)	<0.001	−2.80	0.06	0.03, 0.14
Pupil distance (mm)	0.04	−0.10	0.90	0.82, 0.99
Best corrected visual	0.02	−5.05	0.006	0.00, 0.51
acuity (logMAR)				
Cylindrical refractive	<0.001	0.90	2.45	1.45, 4.14
power (Dpt)				
Refractive Error (Dpt)	<0.001	−0.59	0.58	0.44, 0.70

If steep cornea/keratoconus was defined as a corneal refractive power of 49+ diopters or of 50+ diopters, the study groups were statistically too small for a meaningful multivariate analysis.

If the corneal refractive power was calculated assuming a refractive index of the cornea of 1.3375 instead of 1.3315, the mean refractive power of the cornea in the flattest meridian was 43.9±1.5 diopters (median: 43.9 diopters; range: 37.2 to 49.3 diopters) and it was in the steepest meridian 44.8±1.5 diopters (median: 44.8 diopters; range: 37.7 to 52.8 diopters). Using the corneal refractive index of 1.3375, a steep cornea/keratoconus (defined as a corneal refractive power of 48+ diopters in the steepest corneal meridian) was detected in 71 subjects (prevalence rate: 2.2±0.3% (95%CI: 1.7, 2.8), a steep cornea/keratoconus as corneal refractive power of ≥49 diopters in 23 subjects (prevalence rate: 0.7±0.2% (95%CI: 0.4, 1.0), and a steep cornea/keratoconus as corneal refractive power of ≥50 diopters in 5 subjects (prevalence rate: 0.2±0.1% (95%CI: 0.002, 0.3).

## Discussion

Steep cornea/keratoconus defined as corneal refractive power of 48+ diopters had a prevalence of 0.9±0.2% among Chinese aged 50+ years and living in Greater Beijing. In multivariate analysis, the presence of steep cornea/keratoconus was significantly associated with shorter axial length, smaller interpupillary distance, higher cylindrical and myopic refractive error and lower best corrected visual acuity. It was not significantly associated with any systemic parameter tested, including age, gender, body height and body mass index, consumption of alcohol and smoking, psychic depression and cognitive function, level of education and self-reported income, quality of life, blood pressure and blood concentrations of lipids, creatinine, glucose and C-reactive protein. A steep cornea/keratoconus defined as corneal refractive power of >49 diopters was detected in 6 (0.2%) eyes, and a steep cornea/keratoconus defined as corneal refractive power of >50 diopters was detected in 2 (0.06%) eyes.

Since the present study is the first population-based investigation on the prevalence of steep cornea/keratoconus in a population outside of rural central India, the results of our study cannot directly be compared with findings of other studies. Most investigations on the frequency of keratoconus examined the relative frequency of keratoconus as reason for penetrating keratoplasty or assessed the prevalence of the diagnosis of keratoconus in the Medicare population of the U.S.A [10], [16]. Nielson and colleagues estimated the prevalence and incidence of hospitalized patients with keratoconus in Denmark by analyzing data extracts from the National Patient Registry under the National Board of Health (which covers the entire Danish population) [9]. They found a prevalence of keratoconus of about 86 patients per 100,000 residents and an incidence of 1.3 patients per 100 000 residents per year. This figure of 86/100,000 (or 0.086%) is markedly lower than the prevalence rate of steep cornea/keratoconus of 0.9% in our study population. The reason for the discrepancy between the studies is that the hospital-based investigations on the number of patients undergoing surgical treatment for keratoconus refer to a minority of all subjects with keratoconus. Since, however, the early to medium advanced stages of keratoconus are usually treated by spectacles or hard contact lenses [1], [2], the hospital-based studies on the prevalence of surgically treated keratoconus may only show the minimum of the prevalence of the disorder in the general population.

In the Central India Eye and Medical Study CIEMS, the prevalence of steep cornea/keratoconus defined as a corneal refractive power of ≥48 diopters (and using a corneal refractive index of 1.3375) was 2.3±0.2% (95%CI: 2.0, 2.6) [11]. That figure is almost identical to the prevalence rate in our study (2.2±0.3% (95%CI: 1.7, 2.8)), if the same corneal refractive index (1.3375) was used. Considering the marked differences in the study populations between the CIEMS with a markedly rural and almost tribal population with a low body mass index (19.7±3.4 kg/m^2^) and the Beijing Eye Study with a markedly more urban population with an average body mass index of 25.6±3.8 kg/m^2^, one may infer that the prevalence of steep cornea/keratoconus may not show marked inter-ethnic differences.

In the present study, presence of steep cornea/keratoconus was significantly associated with shorter axial length, smaller interpupillary distance, higher cylindrical and myopic refractive error and lower best corrected visual acuity. Similar results were obtained in the CIEMS, in which the prevalence of steep cornea/keratoconus was significantly (multivariate analysis) associated with higher myopic refractive error (*P* = 0.004), and in which the presence of a steep cornea/keratoconus was not significantly associated with alcohol consumption (*P* = 0.99) or smoking (*P* = 0.08) nor with questions relating to the psychiatric status. In contrast to the present study, steep cornea/keratoconus in the CIEMS was additionally associated with lower body height (*P*<0.001) and lower level of education (*P* = 0.03). In previous studies, associations between keratoconus and Down syndrome, Leber’s congenital amaurosis, and mitral valve prolapse were reported [1]–[5]. Since the number of patients with these relatively rare diseases is too small to be assessed in a population-based study, we could not perform a statistical analysis of a potential association between keratoconus and these disorders.

Interestingly, in the multivariate analysis, the subjects with steep cornea/keratoconus did not vary from the subjects with normal keratometric readings in the answers to questions referring to their psychiatric status, such as “I feel pleasant”, “I feel like crying”, “I feel that difficulties are piling up, so that I can’t overcome them”, “I am happy”, and “Have you ever made an attempt at suicide”. A similar result was obtained in the CIEMS [11]. It may contradict hospital-based reports in which patients with keratoconus have been described to exhibit some abnormal behavior or psychiatric abnormalities [17]. In a parallel manner, prevalence of steep cornea/keratoconus was significantly associated neither with quality of life nor with the level of education after adjustment for region of habitation. It may suggest that the presence of a steep cornea/keratoconus did not have a major impact on career development. In the present study, prevalence of steep cornea/keratoconus was neither correlated with dry eye feeling, what may serve as a relative surrogate for ocular allergies and indirectly for eye rubbing. Previous studies had suggested that eye rubbing and ocular allergies may be risk factors for the development of a keratoconus, a hypothesis which was not fully supported by the findings from our study [17].

In the present study, the prevalence of steep cornea/keratoconus was not related with smoking (history of smoking: 18.5% in the steep cornea/keratoconus group versus 33.4% in the non-steep cornea/keratoconus group; *P* = 0.09). Again, it is in agreement with the CIEMS, however not in agreement with a recent hospital-based study by Spoerl and colleagues, in which a significant correlation was reported between non-smokers and keratoconus (*P*<0.001) [18]. One of the reasons for the discrepancy between the studies may be the difference in the study design (population-based versus hospital-based).

There are limitations of the present study. First, a major concern in any prevalence study is nonparticipation. The Beijing Eye Study 2011 had a reasonable response rate of 78.8%, however, differences between participants and non-participants could have led to a selection artifact. Second, the best technique to detect also minor forms of keratoconus is corneal topography. Due to the limitations of a population-based study, however, the method of corneal topography could not be included into the study design. There is, therefore, the possibility that the diagnostic criterion of an anterior corneal refractive power of ≥48 diopters as measured by keratometry may have resulted in false positive results (spherical corneas with high keratometric readings and scarred corneas with high keratometric readings) and in false negative results (eyes with mild keratoconus). Third, in a similar manner, another limitation is the definition of steep cornea/keratoconus as a corneal refractive power of 48+ diopters. One has, therefore, to keep in mind that the reported prevalence figures and the associations refer to this definition.

In conclusion, in adult Chinese aged 50+ years and living in Greater Beijing, a steep cornea/keratoconus defined as corneal refractive power of 48+ diopters had a prevalence of 0.9±0.2%. Prevalence of steep cornea/keratoconus was significantly associated with the ocular parameters of shorter axial length, smaller interpupillary distance, higher cylindrical and myopic refractive error and lower best corrected visual acuity, however, with none of the systemic parameters tested.
